# Vitamin D and Kidney

**DOI:** 10.1155/2013/164103

**Published:** 2013-12-26

**Authors:** Hulya Taskapan, Ibrahim Sahin, Paul Tam, Tabo Sikaneta

**Affiliations:** ^1^Internal Medicine, Faculty of Medicine, Inonu University, 44280 Malatya, Turkey; ^2^Endocrinology Department, Internal Medicine, Faculty of Medicine, Inonu University, 44280 Malatya, Turkey; ^3^Nephrology Department, The General Hospital, Toronto, ON, Canada M1P 2V5

After absorption from the gut or entry into the circulation from the skin, vitamin D is hydroxylated to 25(OH)D by 25-hydroxylase enzyme in the liver and then to 1*α*,25(OH)D_3_ (or calcitriol) by 1*α*-hydroxylase enzyme in the kidney and in many cells.

The hormonal or active form of vitamin D, that is, 1,25(OH)_2_D_3_ acts through a nuclear receptor (VDR) to carry out its many functions. The receptor has been recognized in a wide variety of tissues such as in osteoblasts, distal renal tubular cells, parathyroid gland cells, skin keratinocytes, lymphocytes, enterocytes, prostate, colon, pituitary gland, and ovaries. Many tissues in the body, *express 1-hydroxylase* and synthesize 1,25(OH)_2_D_3_ locally.

Vitamin D has at least 2 distinct groups of functions. One is classically endocrine functions regulating PTH secretion and hence its own production. The other is autocrine functions (or paracrine): the cells or tissues concerned make and degrade active vitamin D locally to regulate their own proliferation and differentiation. Vitamin D supplementation can affect many aspects of health because *function*.

Many epidemiologic studies have shown that chronic vitamin D deficiency *is associated with an increased risk of diseases such as dementia,* hypertension, multiple sclerosis, rheumatoid arthritis, cancer of the colon, prostate, breast, and ovary, and types 1 and 2 diabetes.

In the general population and especially in patients with chronic kidney disease (CKD) has been reported. The reasons for hypovitaminosis D in CKD are not known but likely include reduced oral intake of vitamin D because of dietary restrictions and/or anorexia.

In this special issue about vitamin D and kidney, Nordenström evaluated vitamin D status in patients operated for primary hyperparathyroidism (PHPT) and compared the patients from southern and northern Europe. These authors found that postmenopausal women with PHPT from Spain had lower preoperative levels of 25(OH)D and more severe PHPT Swedish *patients, and* patients with PHPT and vitamin D deficiency gained more bone density at some sites one year after parathyroidectomy.

The effects of vitamin D on *gentamicin-induced* acute kidney injury *in an experimental* rat model were investigated *by *Hur et al. The authors reported that vitamin D does not *seem to affect histological* findings although some beneficial effects via Renin Angiotensin System and a promising effect on antioxidant system.

Cardiometabolic risk factors related to vitamin D and adiponectin in obese children and adolescents were evaluated by Kardas et al. The authors showed that lower vitamin D and adiponectin levels were strongly associated with metabolic risk factors and obesity in children and adolescents.

Kidney disease was found to be a major risk factor for vitamin D deficiency population study of both *hospitalized and non-admitted patients*. Significant independent predictors of vitamin D deficiency in inpatients and outpatients of a nephrology unit were retrospectively studied by Bentli. These authors showed that vitamin D deficiency is an important problem in both inpatients and outpatients of nephrology. Monitoring serum 25(OH)D concentrations regularly and replacement of vitamin D are important.

Finally, Yildirim et al. report their findings on the association between inflammatory markers (C-reactive protein, erythrocyte sedimentation rate, and leukocyte count) and the presence of CKD in vitamin D-deficient patients sedimentation rate, and leukocyte count, in vitamin D deficient patients with and without chronic kidney disease.

